# RhoC Regulates Cancer Stem Cells in Head and Neck Squamous Cell Carcinoma by Overexpressing IL-6 and Phosphorylation of STAT3

**DOI:** 10.1371/journal.pone.0088527

**Published:** 2014-02-12

**Authors:** Mozaffarul Islam, Smita Sharma, Theodoros N. Teknos

**Affiliations:** 1 Department of Otolaryngology-Head and Neck Surgery, The Ohio State University Wexner Medical Center, Columbus, Ohio, United States of America; 2 Comprehensive Cancer Center, The Ohio State University Wexner Medical Center, Columbus, Ohio, United States of America; Johns Hopkins University, United States of America

## Abstract

In this study we investigated the correlation between RhoC expression and cancer stem cells (CSCs) formation in head and neck squamous cell carcinoma (HNSCC). The inhibition of RhoC function was achieved using shRNA. The expression of stem cell surface markers, ALDH and CD44 were significantly low in two RhoC depleted HNSCC cell carcinoma cell lines. Furthermore, a striking reduction in tumorsphere formation was achieved in RhoC knockdown lines. The mRNA expression of RhoC in RhoC knockdown adherent and tumorspheres are dramatically down regulated as compared with the scrambled control. The mRNA expression of stem cell transcription factors; nanog, oct3/4 (Pouf1), and sox2 were significantly depleted in RhoC knockdown clones. Further, the phosphorylation of STAT3^ser727^, and STAT3^tyr705^ were significantly down regulated in RhoC knockdown clones. The overexpression of STAT3 in RhoC knockdown did not show any change in expression patterns of either-STAT3^tyr705^ or stem cell transcription factors, signifying the role of RhoC in STAT3 activation and thus the expression of nanog, oct3/4 and sox2 in HNSCC. The expression of Inter leukin-6 (IL-6) in RhoC knockdown HNSCC cell lines was dramatically low as compared to the scrambled control. Further, we have shown a rescue in STAT3 phosphorylation by IL-6 stimulation in RhoC knockdown lines. This study is the first of its kind to establish the involvement of RhoC in STAT3 phosphorylation and hence in promoting the activation of core cancer stem cells (CSCs) transcription factors. These findings suggest that RhoC may be a novel target for HNSCC therapy.

## Introduction

Head and neck squamous cell carcinoma (HNSCC) is among the top ten fatal cancers worldwide [1], [2]. Moreover, as reported by the American Cancer Society, approximately 41,380 new cases will be diagnosed in the year 2013, out of which about 19% of patients are likely to die due to the disease in the same year [3]. The survivors face secondary manifestations of the disease resulting in a prolonged and extensive treatment. This is exacerbated by the fact that the disease shows a high frequency of re-occurrence. As a result, HNSCC patients face a long battle against the disease causing great economic and emotional burden [4]. Consequently, a report by Brown *et al* (2002) cites HNSCC among the eight most expensive cancers in the Medicare program [5].

The unusually high morbidity and mortality rate is due to the malignant nature of HNSCC and its widespread occurrence in most head and neck cancers. Therefore, it is not uncommon to find metastasis to lymph nodes of the neck region leading to loco-regional failure (most frequent) followed by pulmonary and bone metastasis [6], [7]. As a result, patients with HNSCC show poor prognosis and a five year survival rate of only 50–60% [3]. Thus, there is a great need to understand the genetic mechanisms regulating the malignancy of HNSCC and use them to design better treatment strategies that can prevent metastasis and re-occurrence.

RhoC is a member of the well characterized Rho family of GTPases that are involved in a wide range of cellular activities including intracellular signaling, cytoskeletal organization, cell proliferation and the regulation of gene expression [8]. Interestingly, the Rho genes belong to the Ras superfamily, many of which have been identified as oncogenes [9], [10]. Although very few genetic mutations are observed in the RhoC gene, it is reported to be over-expressed in many forms of invasive carcinomas including HNSCC [11], [12]. Specifically, studies in all types of cancers where RhoC expression was analyzed revealed a very strong correlation between greatly increased expression and metastasis. Moreover, when RhoC function is inhibited *in vitro*, it results in a strong reduction of cell invasion and motility [13]. Interestingly, *in vivo* studies of tumorigenesis in RhoC knockout mice show tumors with a greatly reduced ability to metastasize to the lungs [10]. Altogether, these studies strongly suggest RhoC is a pro-metastasis oncogene that plays a significant role in transforming non-invasive tumor cells into an invasive phenotype.

The study of RhoC function focuses mainly on its role in the reorganization of the cytoskeleton by inducing the formation of stress fibers and focal adhesion, which are critical steps toward changing cells into motile and invasive forms [14]. However, the process of metastasis by cancer cells is a complex and multistep process which is accompanied with the increased expression of genes that enhance motility and invasiveness and a selective down-regulation of genes that inhibit this process. The prevalence of RhoC in a wide range of invasive carcinomas and its function as a signaling GTPase suggests it can regulate other pathways which are involved in the transformation of tumor cells into a metastatic phenotype.

Cancer stem cells (CSCs) are a subpopulation of undifferentiated tumor cells within a tumor mass which are capable of self-renewal. They also exhibit strong resistance to chemo-radiation therapy. Furthermore, CSCs can persist in tumors as a discrete sub-population which can relapse and metastasize by forming new tumors [15], [16]. These unique properties play a significant role in cancer cell survival leading to recurrence. Importantly, they can also be a source of cells with an invasive phenotype, thus playing a key role in metastasis.

A sizable number of studies have been conducted on the presence and role of CSCs in head and neck cancer which has been reviewed comprehensively by Minnelli and Gallo (2012) [17]. One study of primary tumors in head and neck cancers using the markers CD44 and ALDH identified a subset of tumor cells that unique CSCs properties including self-renewal, capacity to form new tumors in mice and exhibited high metastatic potential (Prince *et al* 2007, Davis *et al*, 2010, Clay *et al* 2010) [16], [18], [19]. Interestingly, the ALDH positive tumor cells exhibited more tumorigenic potential when compared to CD44 positive cells [19]. Moreover, Chen *et al* (2010) [20] reported primary tumors with a higher expression of CD44 and ALDH, which attributes well with the poor prognosis in HNSCC patients. Based on these studies, it can be concluded that CSCs can play a significant role in metastasis of HNSCC; thus suggesting a need to understand the molecular mechanisms that regulate them.

CSCs have been also identified and characterized in HNSCC cell lines where they have been shown to play an important role in the epithelial-mesenchymal transition (EMT) [21]. In our study, we have analyzed the effect of RhoC inhibition on the functional characteristics of cancer cells that exhibit CSC-like features in HNSCC cell lines. We show that inhibiting RhoC activity in HNSCC cell lines results in a significant reduction in the cell population expressing CD44 and ALDH, markers of CSCs in head and neck tumors.

Furthermore, we also characterized the molecular mechanism by which RhoC regulates the growth and self-renewal of CSCs. Our results show that RhoC can significantly lower the expression levels of the core stem cell transcription factors, nanog, sox2 and oct3/4. We further show that this is achieved by reducing levels of phosphorylated STAT3 via the IL-6/JAK signaling pathway. This study is the first of its kind that attempts to dissect the role of the RhoC GTPase signaling pathway in the regulation and activation of stem cell transcription factors. (These factors promote and maintain tumor cells with stem cell-like properties in head and neck cancer).

Therefore, based on our findings we conclude that RhoC is an important oncogene that is needed for the maintenance and propagation of CSCs in HNSCC.

## Materials and Methods

### Cell culture

Head and neck squamous cell carcinoma cell lines UM-SCC-1 and -47 (University of Michigan Squamous Cell Carcinoma) were procured from the University of Michigan. These are well characterized lines derived from patients with T2N0 of floor of the mouth and T3N1 of base of the tongue respectively [22], [23]. Cell lines were grown as described earlier [13].

### Lentivirus transduction, infection and selection of positive RhoC knockdown clones

In order to get stable RhoC knockdown clones from the aforementioned cell lines we used RhoC knockdown and scrambled sequence control constructs with GFP tagged and puromycin resistance sites as described in our previously published work [13]. Selection of RhoC knockdown stable clones was achieved using 2.0 and 1.6 µg/ml puromycin for UM-SCC-1 and -47 respectively. These clones were further sorted by flow cytometry to get the maximum number of GFP positive cells, which were also used in subsequent studies.

### Transient transfection of RhoC-siRNA

Because the emission spectra of GFP tagged with RhoC shRNA was the same as the emission spectra of the fluorescence antibody used to identify ALDH positive cells, the transfected RhoC-shRNA/GFP knockdown clones generated from UM-SCC-1 and -47 cell lines could not be used to identify and sort the CSC sub-population. Instead, knockdown of RhoC was achieved transiently in both UM-SCC-1 and -47 using RhoC-siRNA (Ambion/Life technologies, USA) and lipofactAMIN 2000 as the carrier. Successful transfection and inhibition of RhoC was demonstrated by the lack of its expression with Western blot analysis using anti RhoC antibody.

### Quantitative reverse transcriptase polymerase chain reactions (qRT-PCR)

Total RNA was isolated by TRIzol reagent (Invitrogen, Carlsbad, CA, USA). Quantitative reverse transcriptase polymerase chain reactions (qRT-PCR) were performed by the Taqman probe system from Applied Bio Systems (Foster City, CA) by using the following products RhoC:Hs00733980_m1, Sox2:Hs01053049_s1, Nanog: Hs02387400_g1 and Oct3/4 (POUF1) Hs01895061_u1. OZ1 and G3PDH were used as the data normalizers. Relative changes in gene expressions were calculated using the 2^−(−Δ)C^_T_ method [24].

### Western blot analysis

Western blot analyses were performed according to the standard protocol. The primary antibodies used were polyclonal RhoC, and p-STAT3^ser 727^, p-STAT3^tyr705^ (1∶1000 dilutions) and αβ Tubulin (as a loading control) (1∶2000 dilutions). These antibodies were the product of Cell Signaling (Cell signaling Technologies, Inc., Boston, USA). After incubation with primary antibodies the membranes were blotted for one hour with secondary HRP-conjugate anti-rabbit antibody (1∶2500) (GE Healthcare Life Sciences, Piscataway, NJ, USA). ECL–super signal system (Thermo Scientific, Rockford, IL, USA) was used for protein visualization.

### Determination of active RhoC or [RhoC-GTP]

[RhoC- GTP] in UM-SCC-1 and -47 scrambled control and RhoC knockdown clones were determined by G-LISA as described earlier [25], using the G-LISA kit (Cytoskeleton Inc., Denver, CO, USA) and following the manufacturer's protocol using RhoC primary antibody from Cell Signaling Inc.

### Enzyme-linked immunosorbent assay (ELISA)

Serum free supernatants from the scrambled control and RhoC knockdown cell lines obtained after 48 hours of incubation were sent for ELISA to the Cytokine Core Lab University of Maryland, Baltimore, MD. Average values of IL-6 (after normalizing with the number of cells from where the supernatants were collected) in terms of pg/ml were depicted in a bar graph.

### Flow cytometry analyses

Flow cytometry analysis for sorting out GFP, ALDH and CD44 positive cells was performed using BD FACS Aria IIU flow cytometer equipped with a 488 nm, 15 mW, air-cooled Argon laser. An ALDEFLOUR kit (Stem cell Technologies, Vancouver, BC, Canada) was used for ALDH staining. FACS analysis was performed at flow lab core facility at The Ohio State University Comprehensive Cancer Center.

### Tumorspheres formation

For tumorsphere formation assays we sorted out the GFP positive cells and used them for all subsequent experiments. Tumorspheres from the scrambled control and RhoC knockdown lines were obtained by growing an equal number of cells (1×10^6^) on ultra low attachment plates in keratinocyte serum free media supplement with EGF 20 ng/ml, bFGF 20 ng/ml, insulin 100 ng/ml, and hydrocortisone 400 ng/ml. For propagating the spheres to the next generation, spheres were filtered out on a 40 µM cell strainer and briefly trypsinized in a water bath at 37°C. After spinning at 300×g cell pallets were re-suspended in tumorsphere media and plated onto fresh low attachment plates. To determine the efficiency of tumorsphere formation, 500 cells from the scrambled control and RhoC knockdown were plated in 96 well low attachment plates. The numbers of spheres were counted after two weeks of seeding. The ultra-low attachment plates were the product of Corning Incorporated, Corning, NY, USA.

### Statistical Analysis

Statistical analyses (Student's t-test) were performed using Sigma graph pad prism 4 software. The mean was reported with Standard deviation (±SD). Differences were considered to be statistically significant when *p* values were less than 0.05.

## Results

### RhoC expression is significantly reduced in RhoC knockdown HNSCC cell lines

**Figure 1:**
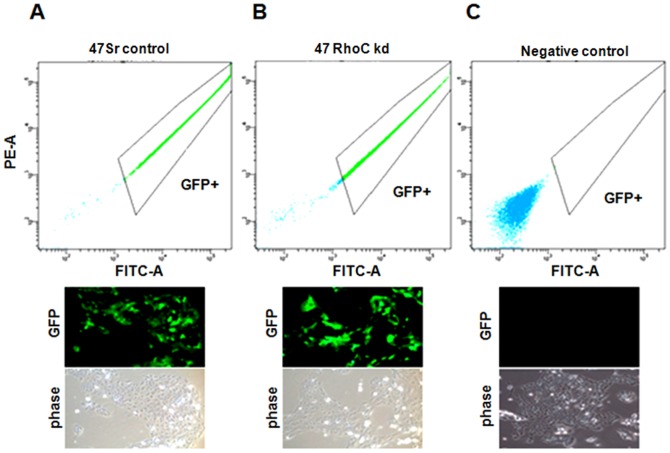
Lentivirus infection and transduction in UM-SCC- cell line. Lentivirus infected cells showing the GFP expression in UM-SCC-47. (A) Histograms of the scrambled control (sr control) and (B) RhoC knockdown (RhoC kd) obtained by flowcytometry. (C) Negative control. Representation of the GFP expression in fluorescence and bright light are shown in the bottom panels. About 90% of cells were GFP positive signifying the successful transduction of shRNA using recombinant lentivirus.

The inhibition of RhoC expression was carried out using small hairpin RNA (shRNA) and the lentiviral transduction and infection methodology as described in the methods section. After lentiviral infection, RhoC knockdown clones were selected using Puromycin (1.6 µg/ml) antibiotic. The number of cells that were successfully infected was analyzed by flow cytometry. This revealed a remarkably low number of non-infected cells (Fig. 1A–C top panel). In addition, fluorescence microscopy of the stable clones shows a strong green fluorescence in the majority of the cells, signifying a high efficiency of lentivirus transduction and infection (Fig. 1A–C bottom panel). These GFP positive cells were further sorted out and re-grown for subsequent experiments. Figure 1C is the negative control showing the parental line without the lentivirus infection. This represents the difference between GFP expressing (infected) and non-GFP expressing cells.

**Figure 2:**
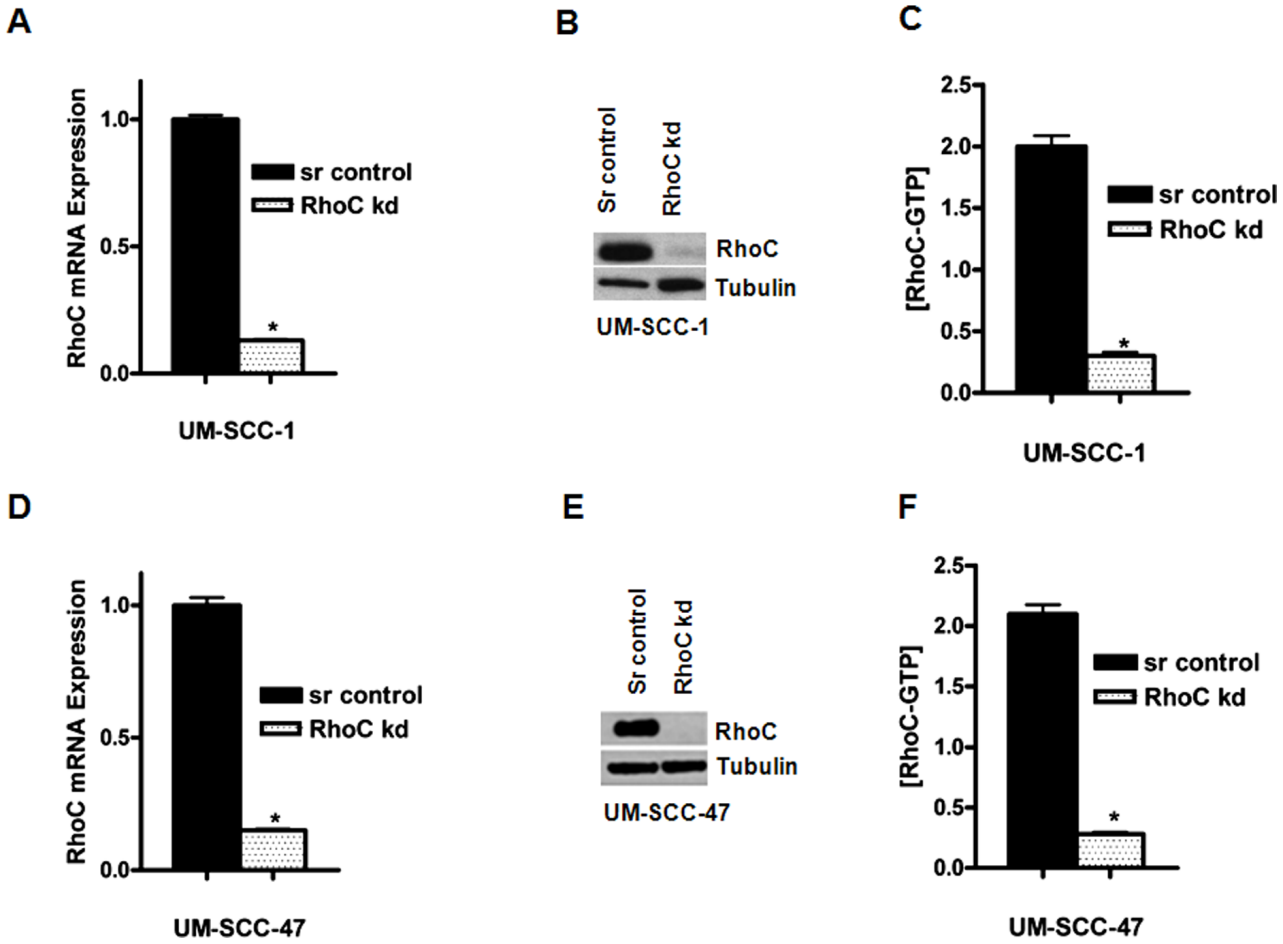
RhoC expression in scrambled control and RhoC knockout UM-SCC-lines. (A and D) The mRNA expression of RhoC obtained by real time RT-PCR; (B and E). Western blot analysis showing RhoC expression in the scrambled control and RhoC knockdown clones (C and F). Bar graph showing the expression of active RhoC [RhoC-GTP] in scrambled control and RhoC knockdown UM-SCC-1 and-47 respectively. A significant decrease in RhoC mRNA, protein and [RhoC-GTP] levels were obtained in the RhoC knockdown cell lines.

We then tested the effectiveness of shRNA in depleting RhoC mRNA expression by real time quantitative PCR (qRT-PCR) in our selected cell lines. As shown in Figure 2, the mRNA expression levels of the RhoC gene in the knockdown clones were significantly low (Fig. 2A&D), while the protein expression was not detectable in the Western blot (Fig. 2B&E). In contrast, sufficient RhoC expression was observed in clones with the shRNA-scrambled sequence control. The relative RhoC mRNA expression in the shRNA-scrambled control and the RhoC knockdown clones was evaluated by quantitative RT-PCR and the C_T_ values obtained were normalized using two housekeeping genes as described in the materials and methods section. A decrease of about 80% of the RhoC mRNA expression was observed in UM-SCC-1 and- 47 knockdown clones as compared to the scrambled control (Fig. 2A&D). In our previous study, we confirmed that only RhoC mRNA expression was inhibited when we used RhoC shRNA constructs made with specific sequences of the RhoC mRNA; furthermore, the expression levels of other Rho proteins were not affected in the UM-SCC cell lines [13]. Thus, we used the same RhoC shRNA constructs for our current study.

Next, we analyzed the RhoC protein expression using Western blot and observed a dramatic depletion of the RhoC protein in RhoC knockdown clones (Fig. 2B&E). Further, the expression of active RhoC [RhoC-GTP] was determined by G-LISA and a similarly low expression of active RhoC was detected in the RhoC knockdown clones of UM-SCC-1 and-47 (Fig. 2C&F). These studies provided clear insight about the “switching off” of RhoC signaling activity by decreasing total levels of RhoC mRNA expression. Further detailed studies on its functional roles in cancer metastasis could be pursued in future research. One of the most basic clinical questions that we addressed at this point is how the knocking down of RhoC function affects tumor cells with stem cell-like properties in head and neck cancer. To address this question, we investigated several biological features of CSCs which include stem cell biomarker analysis and the ability to form tumorspheres. Next, we examined the signaling molecules that maintain the self-renewal properties of CSCs.

### Inhibition of RhoC expression leads to reduced population of ALDH and CD44 positive cells in HNSCC cell lines

In order to identify the CSC population in UM-SCC cell lines, we used the stem cell markers CD44 and ALDH together with fluorescence activator cell sorting (FACS) to separate and count the number of cells expressing them. Moreover, it is important to note that RhoC-siRNA clones of UM-SCC-1 and -47 were used for FACS analysis to determine the ALDH positive cell populations instead of lentivirus infected GFP-RhoC-shRNA clones. This was to avoid the superimposition of GFP fluorescence over the fluorescent labeled ALDH antibody, since their emission spectra overlap.

**Figure 3:**
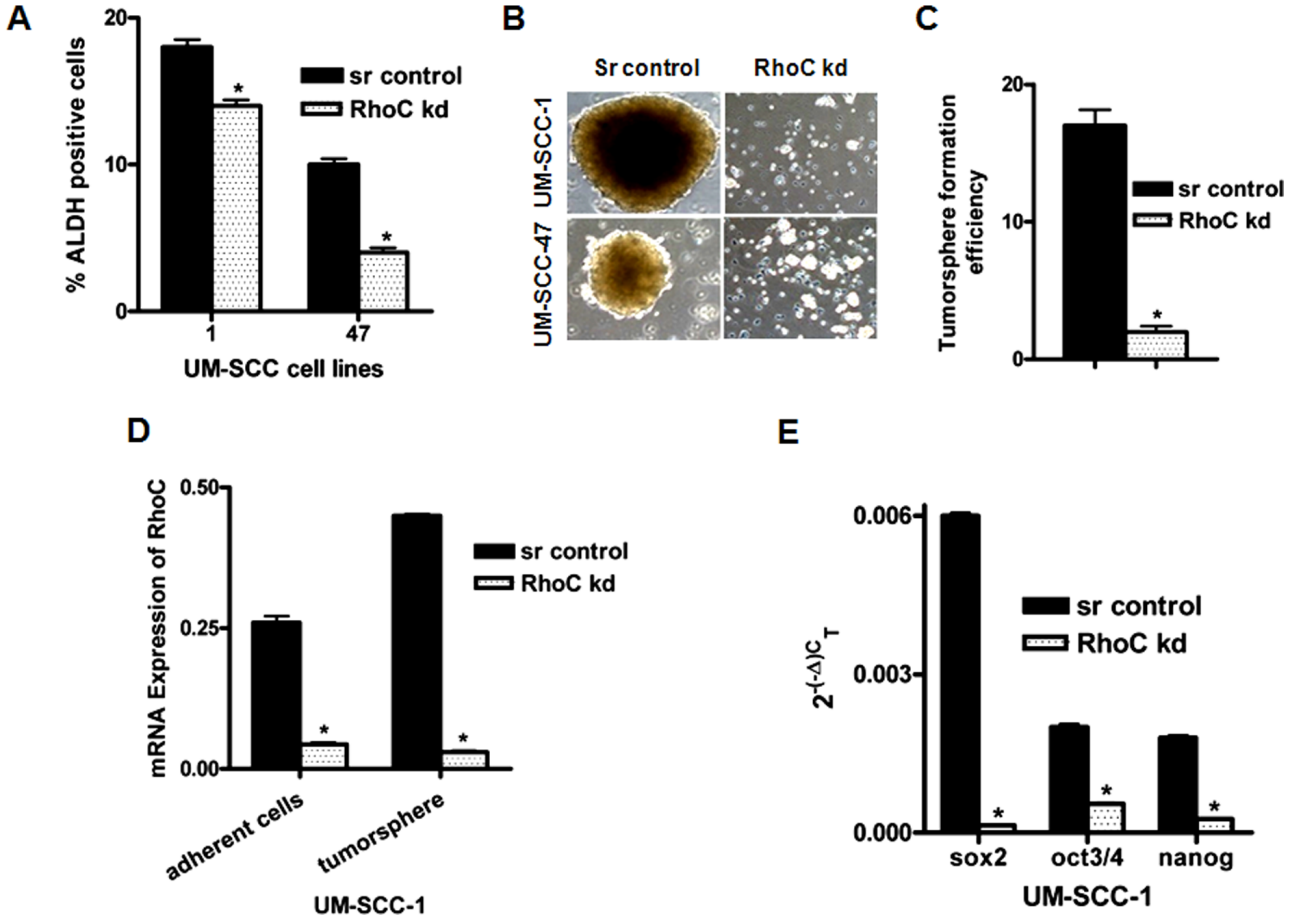
ALDH expression and tumorsphere formation in scrambled control and RhoC knockdown UM-SCC- cell lines. FACS analysis showing the expression of (A) ALDH in the scrambled control and the RhoC knockdown clones. (B) The tumorspheres formation in the scrambled control and the RhoC knockdown UM-SCC-1 and -47cell lines. (C) The representation of tumorspheres formation efficiency in scrambled control and RhoC knockdown UM-SCC-1 obtained in 96 well plates. A significant decrease in ALDH expression was seen. Tumorsphere formation was dramatically low in RhoC knockdown UM-SCC-1 and -47 respectively. (D) A significant decrease in the RhoC mRNA expression was obtained in the RhoC knockdown adherent cell and in the tumorspheres. (E) Stem cell transcription factors, sox2, oct3/4, and nanog showing significant down regulation in their mRNA as revealed by real time RT-PCR in the scrambled control and the RhoC knockdown spheres obtained from UM-SCC-1.

We compared the number of ALDH positive cells in the scrambled control and corresponding RhoC knockdown lines. In the control cell lines, we observed 18% and 10% ALDH positive cells in the total population of UM-SCC-1 and UM-SCC-47 respectively. In contrast, only 13% (UM-SCC-1) and 4% (UM-SCC-47) ALDH positive cells were detected in the corresponding RhoC knockdown lines (Fig. 3A). A similar pattern was observed using CD44, but the percentage of CD44 positive cells was very high. Interestingly, clones with the scrambled sequence control of UM-SCC-1 and -47 showed about 98% CD44 positive cells. In the corresponding RhoC knockdown clones, there were 60% and 41% CD44 positive cells respectively (figure S1). It is worth noting that this was an abnormally high number of CD44 positive cells in both UM-SCC-1 and-47 cell lines (>95%) and therefore CD44 could not be used as a stem cell marker for these cell lines. In addition, our results are similar to earlier reported studies which found CD44 to be abundantly expressed in tumor cells of HNSCC [26].

### RhoC knockdown HNSCC lines are greatly reduced in their capacity to form tumorspheres

Tumor cells with stem cell-like properties are capable of forming floating spheres when grown under non adherent serum free media. Therefore, to further establish the role of RhoC in CSC growth and maintenance, we investigated the tumorsphere formation capabilities in these cell lines. First, an equal number of cells (1×10^6^) from the scrambled control and RhoC knockdown cell lines were seeded on ultra-low attachment plates for observation. We selected five different fields to visualize the number of tumorspheres that had formed. In the scrambled control, there was a high number of tumorspheres with a fewer number of cell aggregates. In contrast, a clear decrease in tumorsphere formation was detected in RhoC knockdown clones generated from both UM-SCC-1 and-47 cell lines. Moreover, it should be noted that well- defined boundaries, a characteristic feature of tumorspheres, are present only in those derived from the scrambled control cells but are evidently absent when derived from the RhoC knockdown cells (Fig. 3B). Instead, what is seen are small cell clusters or aggregates which are devoid of any well-defined outer boundary. Furthermore, these cell clusters did not propagate or form spheres after they were trypsinized and re-plated. In contrast, spheres were easily generated from the control cell lines (scrambled sequence) even after re-plating them up to the fifth generation. We also analyzed tumorsphere formation efficiency in a 96-well plate using 500 cells per well and observed the plate for spheres after two weeks time. A remarkable decrease in the number of spheres (85%) was observed for the RhoC knockdown cells compared to the control cells (Fig. 3C). Similar to the previous tumorsphere assay, cell aggregates were observed from cells derived from the RhoC knockdown cell lines which were quite unlike the well-defined spheres derived from the control clones. We obtained a similar pattern of tumorsphere formation efficiency when the UM-SCC-47 scrambled control and its corresponding RhoC knockdown lines were tested (data not shown). These data suggest that RhoC is important for the growth and maintenance of cancer cells with stem cell-like features in head and neck cancer.

### Analysis of RhoC and stem cell transcription factors expression in adherent and tumorspheres in HNSCC

*Differential expression of RhoC mRNA in adherent cells and tumorspheres*: Next, we analyzed the RhoC mRNA expression in the tumorspheres generated from the UM-SCC-1 cell line and compared it with their corresponding adherent cells using real time RT-PCR. It should be noted that the adherent cells used for the experiments refer to the scrambled control or RhoC knockdown monolayer cells that are grown in standard cell culture substratum. Our result shows that there is an elevated expression of RhoC mRNA in the UM-SCC-1 scrambled control when compared with the RhoC knockdown counterparts (Fig. 3D). Interestingly, the RhoC expression is much higher in the scrambled control tumorspheres when compared to their adherent cell counterparts. This could be due to the presence of a higher concentration of RhoC in tumorspheres where higher numbers of CSCs are localized as compared to the control adherent cell population, which exhibit a mixture of cells with both stem and non-stem cell like features. This further supports our hypothesis that RhoC is required for the maintenance of CSC like features. Our results are in agreement with a similar study on RhoC expression in breast cancer cell lines, where ALDH positive cells exhibited a higher RhoC expression compared to non-ALDH expressing cells [27].

#### Stem cell transcription factors are down regulated in RhoC knockdown tumorspheres

**Figure 4:**
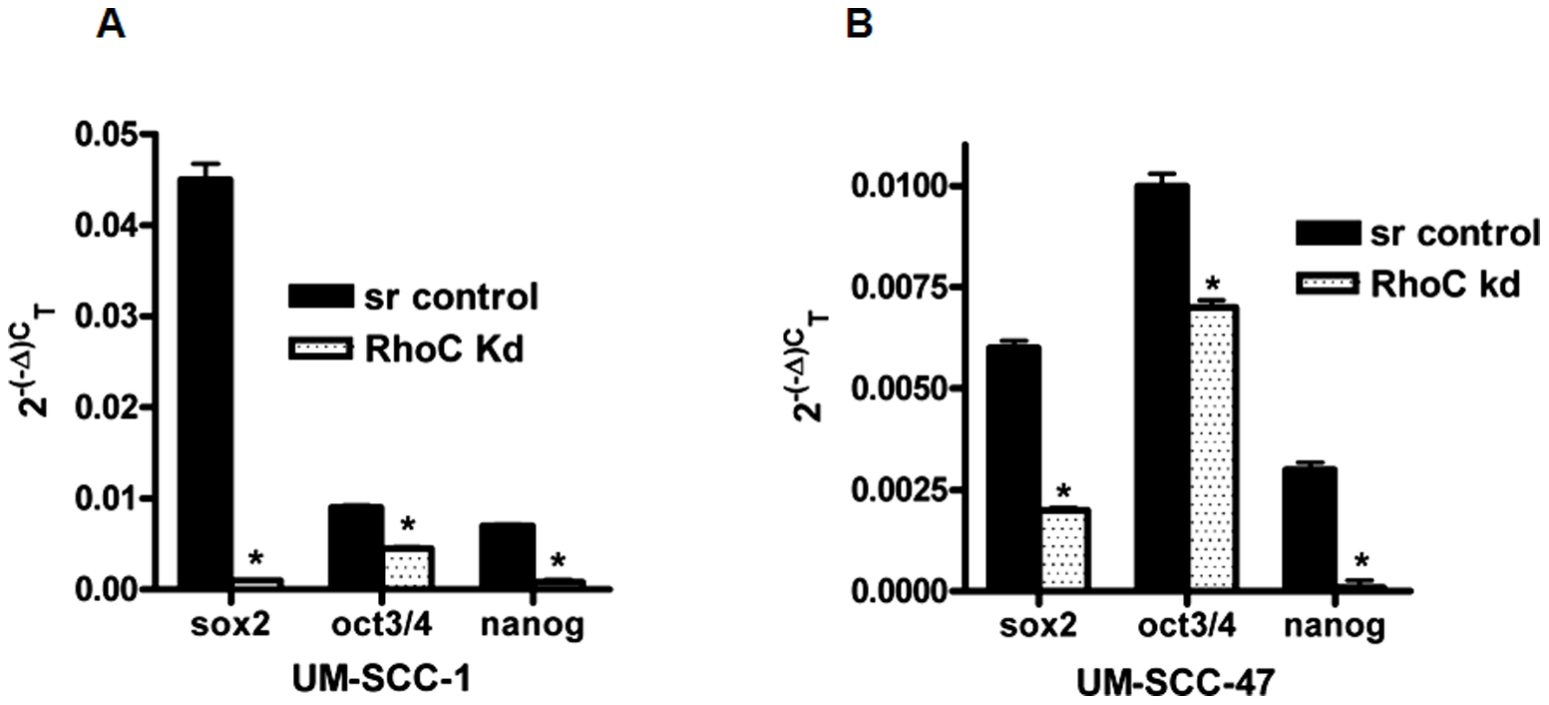
mRNA expression of stem cell transcription factors in scrambled control and RhoC knockdown HNSCC cell lines. Real time RT-PCR showing the mRNA expression of sox2, oct3/4 and nanog in adherent cells of UM-SCC-1(A) and-UM-SCC-47(B). A significant decrease in mRNA expression was observed in stem cell transcriptional factors in the RhoC knockdown HNSCC cell lines.

The growth and self-renewal of stem cells including propagation depend on proper expression of the core stem cell transcription factors nanog, oct3/4 and sox2. Therefore, we analyzed the expression levels of these stem cell transcriptions factors in the RhoC knockdown and scrambled control tumorspheres generated from the UM-SCC-1 cell line by real-time RT-PCR. Interestingly, the expression levels of all three core transcription factors were dramatically reduced in the RhoC knockdown cells when compared to the scrambled control tumorspheres (Fig. 3E). Sox2 was most strongly expressed in the scrambled control tumorspheres, while nanog and oct3/4 were both less strongly expressed but had similar expression levels. However, in the RhoC knockdown counterparts, sox2 and nanog showed the greatest reduced levels followed by oct3/4. Moreover, we also looked at their expression levels in the adherent HNSCC cells lines (scrambled control and RhoC knockdown) from which the tumorspheres were derived. In the UM-SCC-1 scrambled control and RhoC knockdown cell lines, the three transcription factors showed similar levels of expression as observed in the tumorspheres that were derived from them (Fig. 4A). In the UM-SCC-47 scrambled control, oct3/4 had the highest expression followed by sox2 and nanog. Similar to the UM-SCC-1 RhoC knockdown line, nanog showed the greatest reduction when the RhoC expression was inhibited followed by Sox2 (Fig. 4B). In both UM-SCC-1 and -47 RhoC knockdown lines, oct3/4 showed the least amount of reduced expression. Altogether, our results demonstrate that RhoC mediates the propagation and self-renewal of CSCs by regulating the expression level of the core stem cell transcription factors.

### Inhibition of RhoC expression induces down regulation of the STAT3 signaling pathway

**Figure 5:**
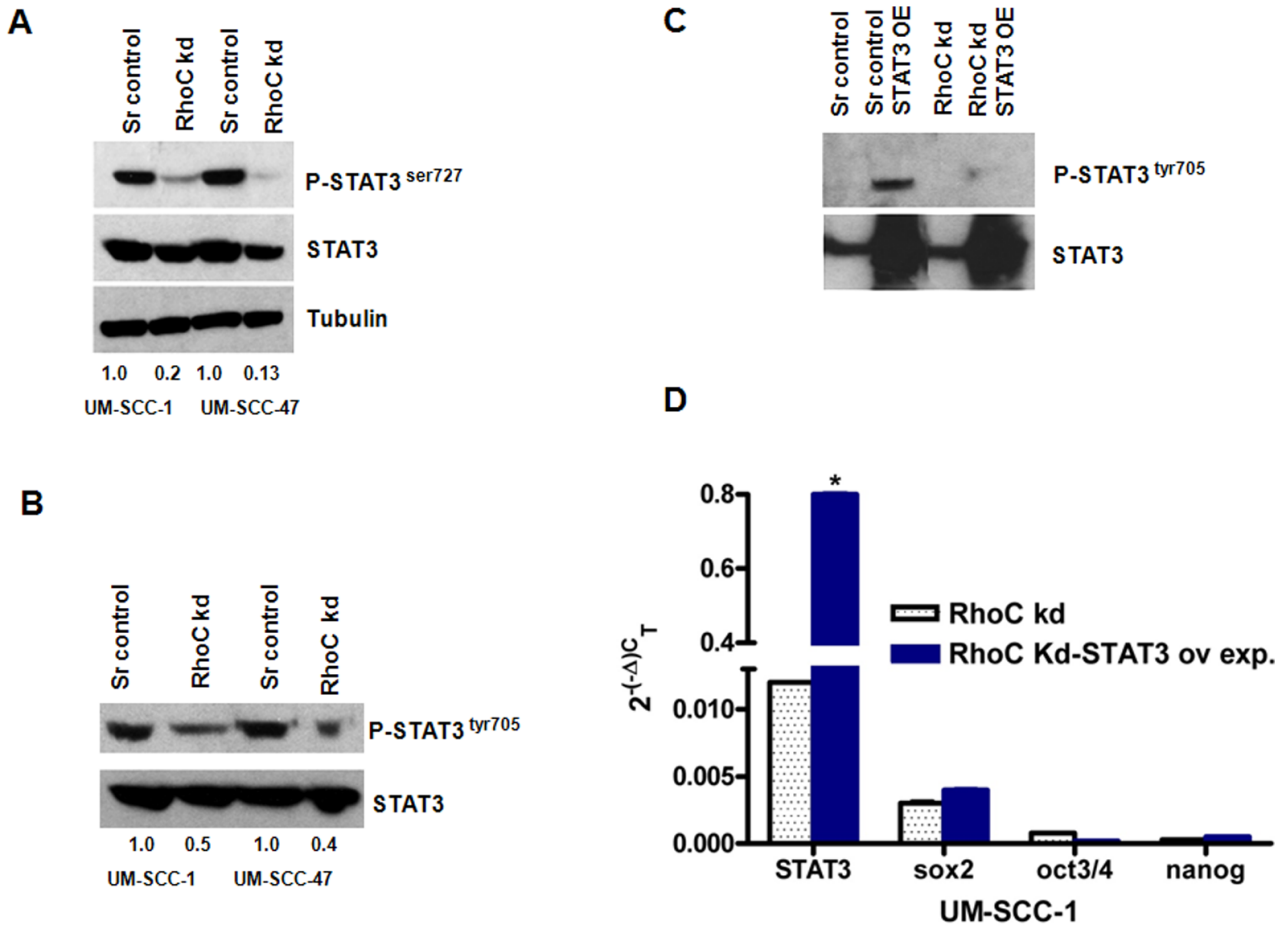
Expression of phospho STAT3 in scrambled control and RhoC knockdown HNSCC cell lines. (A and B) Western blot analysis showing the phosphorylation of STAT3^ser727^ and STAT3 ^tyr705^ in the scrambled controls and the RhoC knockdown UM-SCC-1 and -47 respectively. Normalized value with respect to total STAT3 is given as the numerical values. Remarkable decreases in the phosphorylation of STAT3 were seen in the RhoC knock-down UM-SCC-1 and -47 cell lines respectively. (C) Protein analysis shows the p-STAT3^tyr705^ in the ectopically overexpressed (OE) scrambled control and the RhoC knockdown UM-SCC-1. Phosphorylated form was detected only in the scrambled control when STAT3 was over expressed. A thick band of total STAT3 can be seen in the lower panel, confirming the successful transfection of STAT3 in these clones. (D) Real time RT-PCR showing the expression of STAT3, sox2, oct3/4 and nanog before and after the STAT3 over expression in the RhoC knockdown clone. The mRNA expression of STAT3 was dramatically high after it's over expression, while no significant change in the expression was observed in sox2, oct3/4 and nanog in the RhoC knockdown clone.

Next, we investigated the possible mechanism by which RhoC regulates the expression of the core stem cell transcription factors. The activation of stem cell transcription factors, nanog, sox2, and oct3/4 mediated through Signal Transducers and Activators of Transcription3 (STAT3) signaling pathway is well established [28], [29]. However, the involvement of RhoC in the activation of these transcription factors is not known. Therefore, we analyzed the expression of total and phosphorylated STAT3 (p-STAT3) in the scrambled control and RhoC knockdown HNSCC cell lines. Surprisingly, we observed that while total levels of STAT3 remained about the same in the scrambled control and RhoC knockdown cell lines, p-STAT3 levels was greatly reduced only in the RhoC knockdown clones. Specifically, Western blot analysis revealed reduced phosphorylation of STAT3 protein at ser-727 and tyr-705 residues (Fig. 5A and B). It is of interest to note that phosphorylation at the latter residue is needed for STAT3 to diffuse into the nucleus to bind to promoter elements of STAT3 responsive genes [30], [31]. These results strongly support the idea that the RhoC signaling pathway is required for the activation of STAT3 in HNSCC lines.

To confirm that phosphorylation of STAT3 occurs via the RhoC signaling pathway, we ectopically over-expressed STAT3 in a RhoC knockdown line (UM-SCC-1). The total STAT3 levels in the scrambled control and RhoC knockdown clones when STAT3 was over-expressed exhibited robust bands, signifying a successful transfection of STAT3. Appreciably, the p-STAT3^tyr705^ can only be seen clearly in the STAT3 over-expressing scrambled control cell line. More specifically, in the RhoC knockdown over-expressed STAT3 clone, there was an unremarkable phosphorylation of STAT3 at tyr-705 that could be observed, and was very similar to the expression levels observed in the original RhoC knockdown clones (Fig. 5C).

Furthermore, we analyzed the mRNA expression levels of nanog, sox2, and oct3/4 in the STAT3 over expressed RhoC knockdown clone (UM-SCC-1). As shown in figure 5D, a remarkable increase in STAT3 mRNA expression was observed in this cell line, but the expression of the core stem cell transcription factors, nanog, sox2, and oct3/4 are-unchanged and similar to that observed in the RhoC knockdown lines. These results strongly suggest RhoC is required for the activation of STAT3 and therefore the expression of the core stem cell transcription factors in HNSCC.

### RhoC can regulate STAT3 activation via IL-6 signaling pathway

#### STAT3 activation is regulated by IL-6 in HNSCC

The next question was whether RhoC activates STAT3 directly through a Rho activating kinase or indirectly via another signaling molecule. We analyzed expression levels of Rhotekin and ROCK (Rho-associated coiled-coil kinase), which are two well characterized downstream targets that are activated by RhoC. While we observed the down regulation of both molecules, our immunoprecipitation analysis could not establish an interaction between either Rhotekin or ROCK with STAT3 (data not shown). This strongly suggests that activation of STAT3 by RhoC is regulated via another signaling molecule.

A potential candidate is IL-6, since the activation of STAT3 by IL-6/JAK signaling pathway is well established [32], [33]. Furthermore, there are published studies from our group where increased serum IL-6 levels correlate with advanced head and neck cancer [34]. Another study by Yadav *et al* (2011) [35] reported that IL-6 can induce EMT changes via the JAK-STAT3-SNAIL signaling pathway and thus can promote head and neck cancer metastasis.

**Figure 6:**
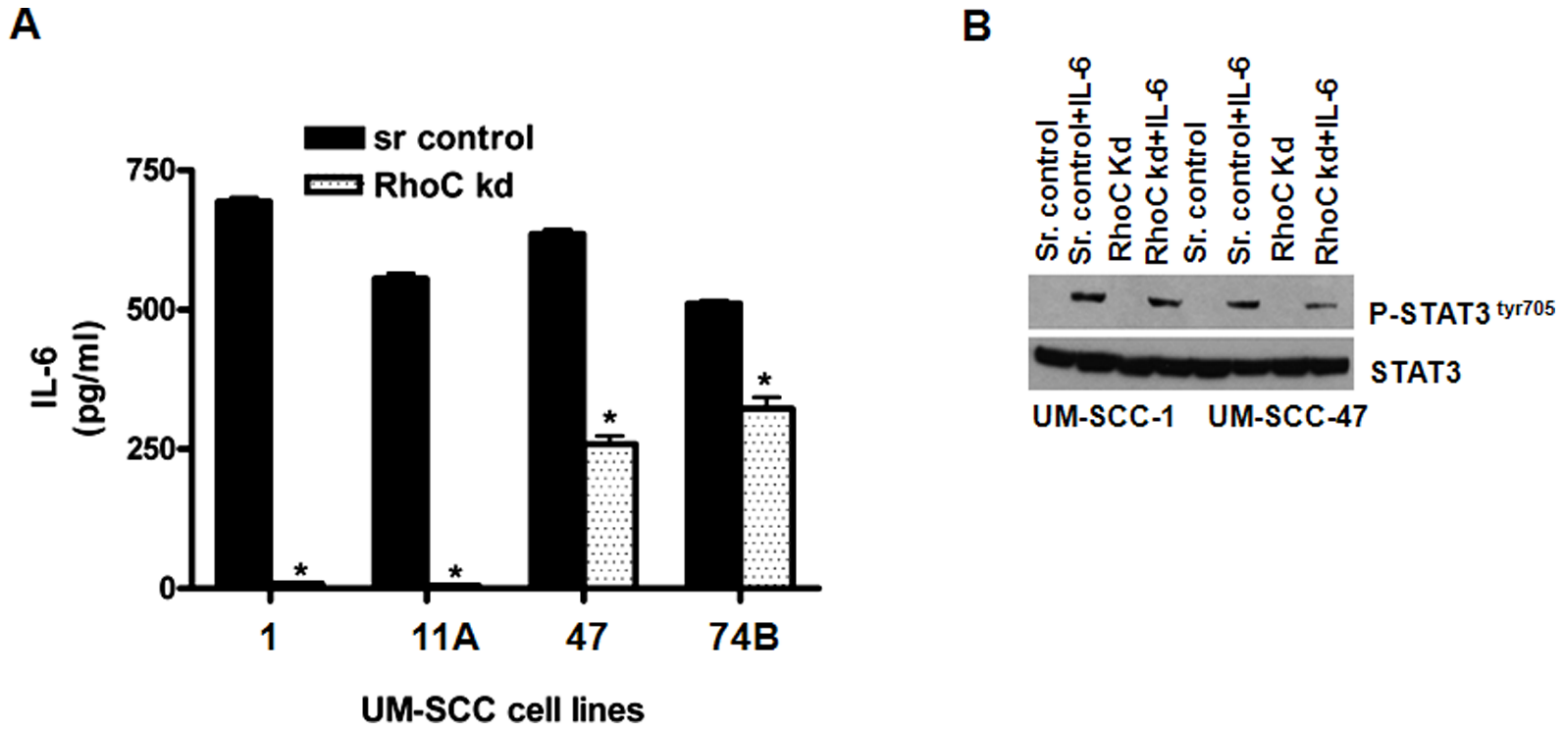
Effect of IL-6 in STAT3 phosphorylation in HNSCC cell lines. (A) A dramatic decrease in the IL-6 expression in the RhoC knockdown cell lines were obtained by ELISA (p≤0.05). (B) Phosphorylation of STAT3^tyr705^ in the scrambled control and the RhoC knockdown cell lines after IL-6 stimulation. Notice IL-6 stimulates STAT3^tyr 705^ phosphorylation even in the RhoC knockdown clones in UM-SCC-1 and -47 respectively.

Therefore, we analyzed IL-6 secreted in serum-free supernatant in four different HNSCC cell lines that express varying levels of RhoC and compared it with their corresponding RhoC knockdown cell lines. Interestingly, we observed notably high levels of secreted IL-6 in all four cell lines tested; the highest was in UM-SCC-1 followed by UM-SCC-47, UM-SCC-11A, and UM-SCC-74B (Fig. 6A). More significantly, IL-6 levels were greatly reduced in all the four cell lines when the RhoC expression was inhibited. Specifically, the IL-6 expression was reduced to almost undetectable levels in UM-SCC-1 and -11A (99%) and strongly reduced in UM-SCC-47 (72%) and -74B (40%) (Fig. 6A). These results provide strong evidence that RhoC can regulate the IL-6 expression in HNSCC.

To establish whether STAT3 is activated by IL-6 in HNSCC lines, we transiently stimulated the UM-SCC-1 and UM-SCC-47 scrambled control and RhoC knockdown clones with IL-6 (50 ηg/ml). The cells were serum starved for an hour and then stimulated with an IL-6 solution for twenty minutes followed by an analysis of phospho- STAT3 using Western blot. As seen in figure 6B, p-STAT3 cannot be detected in any of the serum starved UM-SCC-1 or -47 scrambled control or RhoC knockdown lines. However, when the cell lines were externally stimulated with IL-6, a strong expression of p-STAT3 at tyr-705 could be seen not only in the scrambled control cell lines, but also in the RhoC knockdown counterparts. About 80% and 50% stimulation in STAT3^tyr705^ phosphorylation was detected in the RhoC knockdown UM-SCC-1 and -47 respectively as compared to the scrambled control after IL-6 treatment. This is quite different from the results observed when p-STAT3 could not be detected in the RhoC knockdown UM-SCC-1 cell line even after it was ectopically over-expressed.

These results suggest that when the RhoC activity is inhibited, there is a fall in the IL-6 expression which leads to reduced levels of p-STAT3, while total levels of STAT3 remain similar in both the control and RhoC knockdown. When IL-6 levels were compensated externally, STAT3 is phosphorylated and consequently this promotes an increased expression of the core stem cell transcription factors. In summary, our results demonstrate that RhoC can regulate CSC growth and self-renewal via the IL-6/JAK-STAT3 signaling pathway.

## Discussion

Over-expressed RhoC is one of the established hallmarks for aggressive and metastatic cancers. In addition, a strong correlation between RhoC over-expression and metastasis has been reported in lung [36], melanoma [10], breast [14] and head and neck cancers [12]. Interestingly, the over-expression of RhoC has been reported in inflammatory breast cancer and exclusively in invasive breast carcinoma [11]. In addition, functional studies have shown that RhoC can act as a transforming oncogene when it is over-expressed in human mammary epithelia, converting these cells from immobile into highly motile and invasive cells [11], [14]. In a recent study Rosenthal *et al* [27] reported in their mouse model that active RhoC is one of the key regulators in breast cancer stem cell metastasis. Their study showed that ALDH expressing cells in (breast cancer cell lines) had a much higher metastatic frequency than ALDH negative ones. Thus, a significant number of studies in a wide range of cancers strongly suggest an important role of RhoC in cancer metastasis.

Our own studies on RhoC expression in HNSCC have revealed a strong correlation between RhoC over-expression and lymph node metastasis [12], [37]. In the present study, we investigated the role of RhoC in head and neck cancer stem cell formation and the mechanism of activation of CSC transcription factors.

In our previous studies, we showed that RhoC is constitutively active in several well characterized UM-SCC cell lines [12]. By G-LISA, we analyzed the active form of RhoC in several UM-SCC cell lines (figure S3), and determined that the UM-SCC-1 and -47 lines exhibited considerably high levels of active RhoC (Fig. 2C and F). Therefore, for our current study, we selected these two cell lines to evaluate the role of RhoC in stem cell formation in HNSCC. To better understand the role of RhoC in CSC formation, we inhibited the RhoC function using RNAi coupled with lentiviral transduction and infection strategies to obtain stable RhoC knockdown clones. We established successful inhibition of RhoC mRNA as well as protein expression. We then used these stable clones to elucidate the effect of RhoC inhibition on the head and neck cancer cell population that exhibits stem cell-like features (Fig. 1 and 2). The presence of GFP expression and the knockdown of RhoC expression in UM-SCC-1 has been published in our earlier studies on the effect of RhoC inhibition on cell invasion and tumor formation in mice [13]. As our *in vitro* findings show, the inhibition of RhoC activity caused a significant reduction in cell population that expresses ALDH and CD44 stem cell markers in the RhoC knockdown cell lines. Furthermore, we observed a remarkable reduction in tumorsphere formation and down regulation of core stem cell transcription factors.

Aldehyde dehydrogenase (ALDH) is a well-established marker of CSCs [38], [39]. A high expression of ALDH has been reported in various cancer types including breast and HNSCC and can be used to isolate CSCs with strong metastatic properties [16], [19], [40]. In our current study we show a significant decrease in the ALDH positive cells in the RhoC knockdown head and neck cancer cell lines (Fig. 3A). This implies that the increased expression of RhoC correlates with the higher population of ALDH positive cells, which in turn suggests a significant role of the RhoC in the growth of the CSC population. Furthermore, we have also shown CD44 (another stem cell surface marker) expressing cells decrease in the RhoC depleted head and neck cancer cell lines (figure S2). This is in accordance with the previously published work where a significant overexpression of CD44 was observed in breast and head and neck CSCs [18], [41]. These findings strongly suggest that higher numbers of cells expressing ALDH and CD44 biomarkers are associated with an elevated RhoC expression in HNSCC and hence they can promote and maintain the CSCs in head and neck cancer.

Furthermore, cancer cell lines derived from primary tumors are a heterogeneous population of cells in which a small sub-population exhibit stem cell-like properties. In addition to expressing stem cell biomarkers, these cells also have the ability to survive and grow to form spheres (called tumorspheres) when cultured *in vitro* under non-adherent serum free media [42]. Our tumorsphere assay showed that ALDH positive cells with active RhoC were easily able to form tumorspheres, while the RhoC knockdown counterparts could not form tumorspheres or be cultured for any further generations (Fig. 3B). These results continue to support our hypothesis that RhoC is needed for the growth and maintenance of CSCs in HNSCC.

Moreover, we found a differential pattern of the RhoC expression in the tumorspheres and the adherent monolayer cell culture. Interestingly, the RhoC expression is higher in the tumorspheres formed from the isolated ALDH positive control cells when compared to the corresponding adherent cells (Fig. 3D). These results are in agreement with a similar study in which ALDH positive cells in invasive breast carcinoma line show a higher expression of RhoC GTPase compared to non-ALDH expressing cells. In addition, the same study showed that the cells exhibited higher frequency of metastasis to lungs compared to ALDH negative RhoC knockdown cell lines in the mouse model [27].

The phenomenon of self-renewal and the enrichment property of the stem cells are primarily mediated by the cohort of stem cell transcription factors nanog, oct3/4 and sox2. Nanog, for example, is well-established and required for the acquisition of pluripotency in stem cell [43]. It is also essential in the embryonic stem cell development as well as in preventing cell differentiation in the primitive endoderm [44]. These transcription factors are essential for the development of human embryonic stem cells [44] and many pluripotent cells, including CSCs [45]. In our analysis of the mRNA expression of the core stem cell transcription factors, nanog, sox2, and oct3/4, we found that their levels are greatly reduced in the tumorsphere-like clusters derived from the RhoC knockdown cells. This is in contrast to the tumorspheres of the scrambled control cell lines where strong expression of all three core stem cell transcription factors is observed. Similarly, significant decreases in these core transcription factors were also observed in the adherent cells of RhoC knockdown of HNSCC cell lines (Fig. 4A and B). Therefore, these results suggest that over-expressed RhoC can play a significant role in promoting CSC formation by up-regulating the key stem cell transcription factors in HNSCC (Fig. 3D and E). The next important question we looked at was the signaling pathway by which RhoC regulates CSC growth and maintenance in HNSCC.

To further understand how RhoC regulates the expression of the stem cell transcription factors, we analyzed expression levels of the STAT3, a known activator of nanog [46]. We also examined the STAT3 activation mediated through IL-6 in head and neck cancer. Since the role of RhoC in STAT3 phosphorylation has not been established before, we analyzed the phosphorylation of STAT3^ser727^ and STAT3 ^tyr705^ in the scrambled control and the RhoC knockdown cell lines and showed that there was a decrease in both p-STAT3^ser727^ (80–90%) and p-STAT3^tyr705^ (50–60%) in the RhoC knockdown HNSCC cell lines (Fig. 5A&B). To establish the role of RhoC in STAT3 phosphorylation, we ectopically overexpressed STAT3 in the scrambled control and RhoC knockdown HNSCC cell lines. Phosphorylation of STAT3 ^tyr705^ was observed only in the control cell line, while the RhoC knockdown cell line hardly showed any STAT3 phosphorylation (Fig. 5C). The most probable cause for the lack of phosphorylation of STAT3 in the knockdown line can be attributed to the absence of RhoC activity. Furthermore, there was also no significant change in the expression levels of the core stem cell transcription factors and remained very similar to the RhoC knockdown lines (Fig. 5D). This is noteworthy since it shows that an increasing STAT3 expression ectopically did not make any noticeable changes as long as the RhoC activity was minimal. These findings strongly support a role for RhoC in the activation of STAT3 and hence the growth and self-renewal of CSCs in head and neck cancer.

Next, we examined the probable mechanism by which RhoC activates the STAT3 signaling pathway. The relationship between IL-6 and STAT3 has been reported by Yadav et *al* (2011) [35], where the researchers showed that the over-expression of IL-6 in immortalized oral epithelial cells enhances the phosphorylation of STAT3; this can then induce EMT changes in oral epithelial cells. Furthermore, our group previously reported that IL-6 can be used as a biomarker for predicting the recurrence in and overall survival of a HNSCC patient [34]. In this current study, we found a dramatic decrease in the IL-6 expression in several HNSCC cell lines when the RhoC expression is inhibited (Fig. 6A). It should be noted that these HNSCC cell lines exhibit high levels of constitutively active RhoC and showed a striking decrease in IL-6 levels in their RhoC knockdown counterparts.

To further establish the role of RhoC in STAT3 phosphorylation via IL-6 as an intermediate, we stimulated the serum starved cells with IL-6 and analyzed for STAT3^tyr705^ phosphorylation (Fig. 6B). Surprisingly, the IL-6 stimulation in the RhoC knockdown cell lines phosphorylates STAT3^tyr 705^; this strongly suggests that phosphorylation of STAT3 by RhoC can take place via IL-6. Therefore, IL-6 can be the intermediate molecule between RhoC and the activation of STAT3 in HNSCC. It is worth noting that in serum starved condition p-STAT3^tyr705^ expression could not be detected, and there was a complete absence of protein bands even in the scrambled control. However, when it was stimulated by IL-6, the phosphorylated STAT3 protein could be visualized in both the control and the RhoC knockdown cell lines. The stimulation of IL-6 may trigger the autocrine mechanism of IL-6, resulting in compensating its deficiency in the biological system which would have arisen due to the consequences of the RhoC knockdown. Our results demonstrate that the stimulation of IL-6 in the RhoC knockdown lines can activate the downstream cascade mechanism for the activation of STAT3. To the best of the author's knowledge, this study is the first of its kind to establish the role of RhoC in STAT3 activation via IL-6 in head and neck cancer.

The molecular mechanisms by which RhoC activates the IL-6 expression remain to be explored. It has been reported that the inhibition of NF-κB either by genetic manipulation or by a pharmacological approach severely declined the expression of various cytokines including IL-6. A significant decrease in tumorigenesis in xenograft mouse model was also observed [47]. A number of studies provide strong evidence showing that NF-κB is involved in cellular transformation. In a separate study, Chen *et al* reported that Rhotekin, the gene coding for the Rho effectors, mediates Rho signaling to activate NF-κB signaling, and consequently, the tumors show resistance to apoptotic cell death in gastric cancer [48].

**Figure 7:**
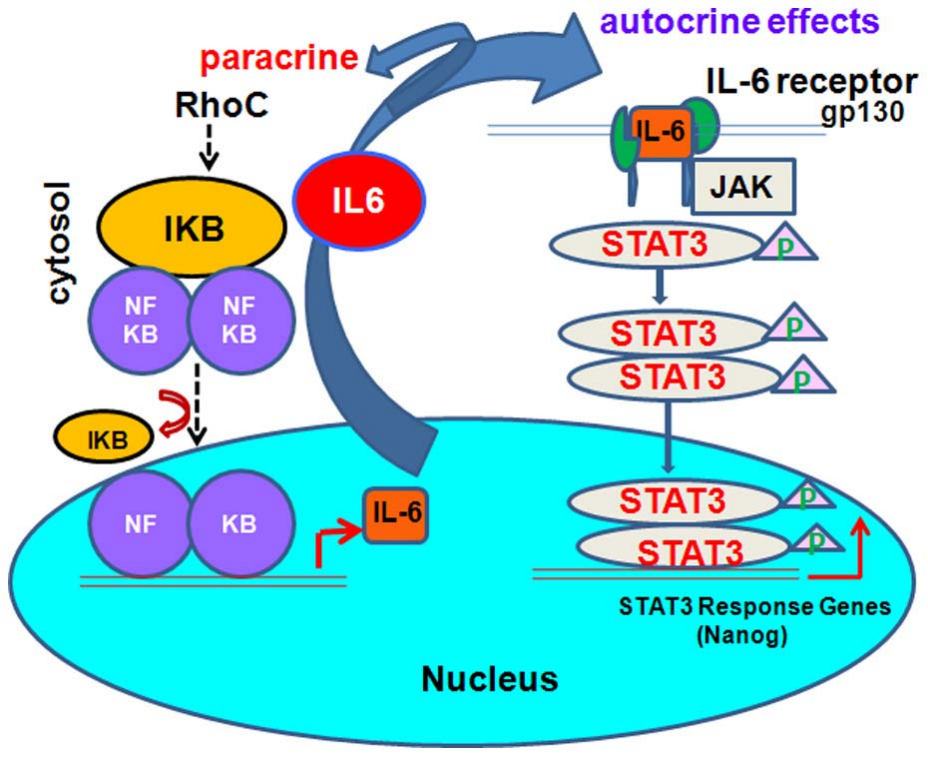
A sketch diagram showing the probable steps involved in nanog activation by RhoC, mediated through the IL-6 and JAK/STAT3 pathway in head and neck cancer metastasis.

Based on our findings, we constructed the signaling pathway by which RhoC regulates CSC growth and self-renewal in head and neck cancer (Fig. 7). RhoC GTPase activates a downstream effector molecule (possibly NF-κB) via a Rho effector kinase and this activated form then turns on the IL-6 gene expression. The mechanism of STAT3 activation by RhoC mediated through IL-6 is most likely through the autocrine system. IL-6 binds to the cell surface protein gp130 and recruits Janus kinases (JAKs), which then phosphorylates STAT3. Phosphorylated STAT3 at tyr-705 is able to dimerize and then diffuse into the nucleus where it binds to the promoter region of nanog to switch on its expression. Nanog is then able to turn on the expression of the additional stem cell transcription factors oct3/4 and sox2, with a resulting increase in tumor cells with stem cell-like properties. In this way, an increased RhoC expression in HNSCC results in a large number of CSCs due to the activation of the core stem cell transcription factors that are needed for their growth and self-renewal. This in turn significantly increases the tumors' ability to grow and metastasize to other body regions.

The implications of these findings provide a fertile area of research in HNSCC. Additional studies are needed to understand how RhoC regulates the IL-6 expression and what additional signaling molecules are involved. A limitation of this study was to obtain an adequate number of tumorspheres from the RhoC knockdown clones for *in vivo* studies. In this study it was not possible to pickup spheres from the RhoC knockdown cell lines as most of them were either aggregates of the cells and not the true spheres or just few in numbers (Fig. 3C). However, the probable solution to overcome this limitation could be to use the primary tumor of the patients expressing different (high and low) levels of RhoC. Tumorspheres can be generated from these differentially expressed RhoC and can then be used for *in vivo* studies.

In conclusion, the findings presented illustrate that RhoC plays an important role in head and neck CSC maintenance and its propagation by modulating the phosphorylation state of STAT3. With additional investigations and ongoing development of RhoC specific inhibitors, this may prove to be an important therapeutic target in the HNSCC patient population. Further, these findings suggest that the inhibition of the RhoC function in HNSCC can diminish the stemness in HNSCC, thus opening new possibilities for future drug therapies targeting this pathway.

## Supporting Information

Figure S1 **RhoC expression significantly depleted by RhoC-siRNA.** (A&B) Western blot analysis shows the expression of RhoC when treated with a different concentration of RhoC-siRNA. As shown at a 100 nM concentration of siRNA, the RhoC expression was completely blocked in both UM-SCC-1 and -47 cell lines. These clones were used for ALDH analysis.
(TIF)

Figure S2 **CD 44 expression was down regulated in Rhoc knockdwon HNSCC lines.** FACS analysis showing the CD44 cells in the scrambled control and the RhoC knockdown UM-SCC-1 and -47 cell lines. As shown here, >95% control cells are CD44 while a significant reduction in RhoC knockdown lines can be seen.
(TIF)

Figure S3 **Active RhoC was dramatically down regulated in RhoC knockdown HNSCC cell lines.** RhoC-GTP was significantly low in the RhoC knockdown clones of UM-SCC-11A and 74B as revealed by G-LISA.
(TIF)
