# Supplementary figures and images for: RhoC Regulates Cancer Stem Cells in Head and Neck Squamous Cell Carcinoma by Overexpressing IL-6 and Phosphorylation of STAT3
Source: PLoS One. 2014 Feb 12;9(2):e88527. doi: 10.1371/journal.pone.0088527 (PMC3922885; doi:10.1371/journal.pone.0088527)

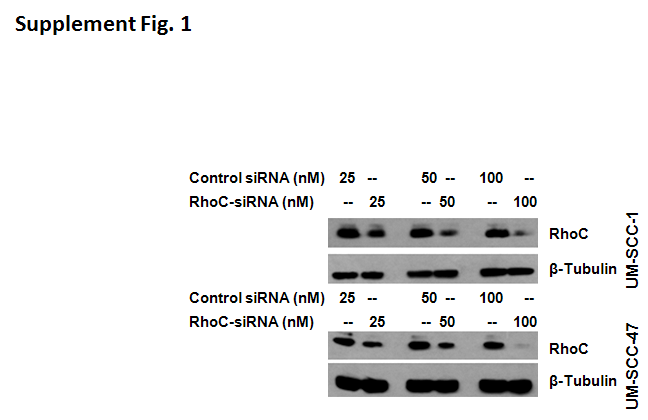

Supplement: Figure S1 — RhoC expression significantly depleted by RhoC-siRNA. (A&B) Western blot analysis shows the expression of RhoC when treated with a different concentration of RhoC-siRNA. As shown at a 100 nM concentration of siRNA, the RhoC expression was completely blocked in both UM-SCC-1 and -47 cell lines. These clones were used for ALDH analysis. (TIF) [file pone.0088527.s001.tif]

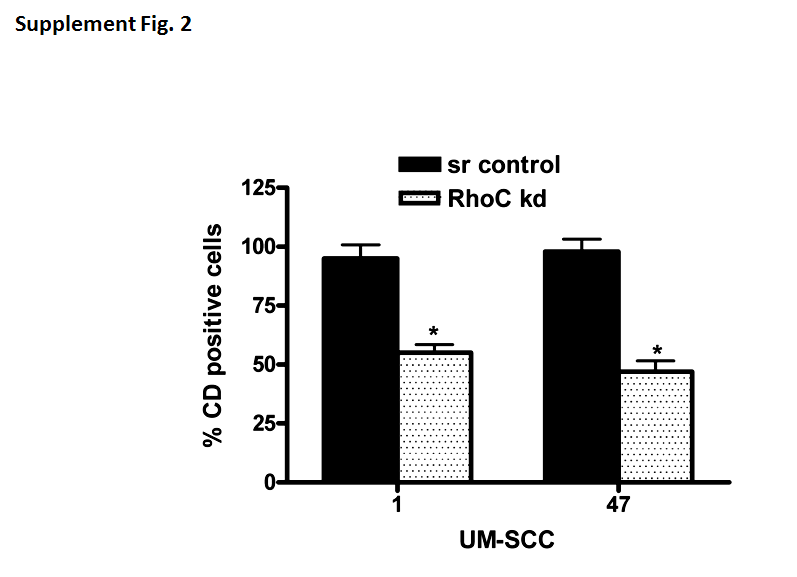

Supplement: Figure S2 — CD 44 expression was down regulated in Rhoc knockdwon HNSCC lines. FACS analysis showing the CD44 cells in the scrambled control and the RhoC knockdown UM-SCC-1 and -47 cell lines. As shown here, >95% control cells are CD44 while a significant reduction in RhoC knockdown lines can be seen. (TIF) [file pone.0088527.s002.tif]

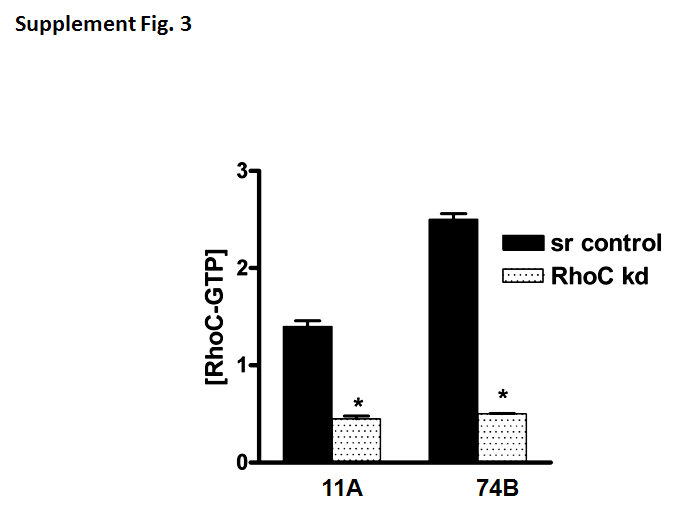

Supplement: Figure S3 — Active RhoC was dramatically down regulated in RhoC knockdown HNSCC cell lines. RhoC-GTP was significantly low in the RhoC knockdown clones of UM-SCC-11A and 74B as revealed by G-LISA. (TIF) [file pone.0088527.s003.tif]
